# Properties of Oaxaca Cheese Elaborated with Ultrasound-Treated Raw Milk: Physicochemical and Microbiological Parameters

**DOI:** 10.3390/foods11121735

**Published:** 2022-06-14

**Authors:** Mariana Huerta-Jimenez, Brissa Herrera-Gomez, Eduardo A. Dominguez-Ayala, America Chavez-Martinez, Juliana Juarez-Moya, Monserrath Felix-Portillo, Alma D. Alarcon-Rojo, Luis M. Carrillo-Lopez

**Affiliations:** 1 Consejo Nacional de Ciencia y Tecnología, Av. Insurgentes Sur 1582, Col. Crédito Constructor, Del. Benito Juárez, Ciudad de México 03940, Mexico; mhuertaj@uach.mx; 2 Facultad de Zootecnia y Ecología, Universidad Autónoma de Chihuahua, Perif. Francisco R. Almada km 1, Chihuahua 31453, Mexico; a307573@uach.mx (B.H.-G.); a310495@uach.mx (E.A.D.-A.); amchavez@uach.mx (A.C.-M.); jjmoya@uach.mx (J.J.-M.); monserrath.felix@uach.mx (M.F.-P.)

**Keywords:** fresh cheese, high-intensity ultrasound, yield, proximal analysis, bacterial control

## Abstract

The effect of ultrasound-treated fresh raw milk upon yield, physicochemical and microbiological quality of Oaxaca cheese was evaluated under a factorial design. The ultrasound frequencies tested were 25 and 45 kHz, during 15 or 30 min. The cheeses made with the ultrasonicated milk (30 min, high-intensity ultrasound, HIU) had greater luminosity without significant changes in hue or chroma, as compared to the controls with no HIU. The yield improved significantly (by up to 2.8 kg/100 L of milk), as the ultrasound treatment time increased. Such cheese yield is attributable to the higher protein content, which was up to 1.5% higher, after sonication. Long-treatment time (30 min) at 25 kHz significantly lowered mesophilic bacteria counts down to limits allowed by current regulations and favors the growth of lactic acid bacteria (LAB) while lowering mold and yeast counts. The absence of *E. coli* and *Salmonella* spp. and the decrease in *S. aureus* counts in Oaxaca cheese were attributed to the mixing of the paste with hot water, inherent to the traditional elaboration process, and to the antagonistic effect of the ultrasound-triggered increased LAB on pathogenic bacteria. Since the artisanal elaboration of Oaxaca cheese does not comply with the current Mexican regulations regarding mesophiles, ultrasound could be a suitable technology to protect its genuine elaboration process with raw milk.

## 1. Introduction

Oaxaca cheese or “quesillo”, as it is traditionally known in Mexico, is an artisanal fresh product (it can contain up to 50% moisture) of soft and spun paste, which is made from raw cow’s milk, using animal natural curd, without any initiator cultures, and is presented in the form of “balls” or strands of different sizes and weights [1,2]. It is the most commercial cheese in Mexico because of its melting properties. Its name is linked to the state of Oaxaca, probable place of origin, where it is traditionally consumed without representing any risk to public health [3,4].

Mexican legislation excludes cheeses made with raw milk, although there are more than 40 varieties of artisanal Mexican cheeses, including Oaxaca cheese. The elaboration of Oaxaca cheese with raw milk does not mean that the product has poor quality, since critical surveillance points during processing guarantees the product innocuousness. For instance, the paste kneading should be carried out with water at 70–80 °C for the strand formation [5]. Unfortunately, scientific studies on Mexican artisanal cheeses are scarce, and there is a lack of standardization and regulations to be observed during the elaboration processes. Thus, more than 40 varieties of Mexican artisanal cheeses are in danger of disappearing [6]. The pasteurization of milk is not always an appropriate option to treat raw milk for cheese production because heat treatment eliminates milk’s beneficial microorganisms (lactic acid bacteria) that determine the final product taste and smell, and could diminish the cheese sensory quality [6]. Oaxaca cheese has milky, buttery, acidic, and fermented flavor notes [7]. Therefore, alternative technologies are required in order to preserve the unique and often unknown characteristics of genuine traditional dairy products, such as Oaxaca cheese.

Emerging non-thermal technologies for food processing are promising because they do not alter the flavor or nutritional value of foods. One of these technologies is high-intensity ultrasound, a physical treatment which leads to the formation of cavitation bubbles that undergo implosive collapse, generating physical and chemical effects in the medium [8,9]. In milk, there are reports on the reduction of particle size and the distribution of its components, modifying the protein structure and improving enzymatic stability [10] by ultrasound application, thus favoring firmness and reducing syneresis in casein gels [11]. The structural changes cause association of fat globules with whey proteins [12]. Moreover, enzymatic activity upon milk components increases, mainly on triglycerides, which leads to the formation of fatty acids, salts, and free radicals that modify the milks’ final pH [12,13,14,15,16]. Additionally, Marquesini et al. [17] reported significant increases in the levels of free fatty acids and oxidation in milk, generating unpleasant flavors (burnt taste) as the intensity and duration of the ultrasound treatment increased (400 W, 24 kHz, 22 mm diameter-probe system). In general, the application of frequencies between 20 and 40 kHz in milk modifies the structure and causes the formation of aggregates due to denaturing and decrease of particle size [18]. Broadly, the greater intensity and longer time of ultrasound treatment in milk (78–430 W, 20 y 24 kHz, 4–15 min, 20, 40 y 69 °C), the more significant reductions in counts of aerobic bacteria, coliforms, and pathogens (*Staphylococcus*), preserving some lactic acid bacteria [19,20]. Nevertheless, the results vary depending on the conditions of the equipment and of the sample. For instance, Chouliara et al. [21] observed an increased count of psychotropic bacteria and in the total viable count during refrigerated storage of thermized (55 °C) and sonicated milk (200 W, 24 kHz, 15–25 °C, 2–16 min). As for the effect of ultrasound in cheese, improvements in gelation and syneresis have been described [18]. In feta cheese, ultrasound (20 min, 20, 40 y 60 kHz, intensity 80%, probe system) accelerated lipolysis and proteolysis during ripening, improving the cheese organoleptic, color, microbial and physicochemical properties [22]. In fresh cheese, higher yielding and reduction in hardness (crumbling) have been described [23], as well as the promotion of yellow tones after 10 min of treatment, and the increase of the pH after 5 min of ultrasound treatment (probe, 400 W, amplitude 100 and 50%). In feta cheese, counts of *E. coli* O157:H7 and *Staphylococcus* spp. are reduced to acceptable levels when the milk is treated with heat and ultrasound combined (thermosonication) [22]. Molds and yeasts are deactivated by this treatment, but no deactivation of *P. chrysogenum* has been observed [22,24].

Regarding Oaxaca cheese, it is necessary to study both, the ultrasound (time, frequency, amplitude, intensity, system) and milk (volume, temperature, total solids) variables, aiming to standardize and improve its fabrication process. In this research, the effect of frequency and time of the ultrasound treatment in raw cow’s milk, upon the physicochemical, microstructural, and microbiological quality of Oaxaca cheese was studied.

## 2. Materials and Methods

### 2.1. Treatment Design

Fresh raw milk was obtained from the Dairy Production Unit of the Universidad Autónoma de Chihuahua. The raw milk came from Holstein cows (30 cows per bulk milk) and was received at 4 °C, 6 h after milking. The milk was treated with high intensity ultrasound (HIU, Intensity = 10–1000 W/cm^2^, Frequency = 20–100 kHz). Before applying the treatments, the physicochemical quality of fresh raw milk was determined in a LactoScan LW milk analyzer by triplicate (Milkotronic Ltd.^®^, Nova Zagora, Bulgaria). The milk parameters that were determined were titratable acidity, fat, protein, lactose, and non-fat solids (NFS) (Table 1). This study was designed as a two-factor completely randomized block experiment with three replicates, as follows: ultrasonic treatment times (control without ultrasound, 15 and 30 min) and HIU frequencies (25 and 45 kHz, 1000 W). For the ultrasound treatment, an Elmasonic^®^ xtra ST 800 H (Singen, Germany) equipment was used, with a minimum operating capacity of 40 L. The amount of treated milk was 40 L, from which 5 L was used to make Oaxaca cheese.

According with the reported treatment times previously used in water bath systems, the longest HIU time was 30 min. Thus, the treatments evaluated were control/25 kHz, 15 min/25 kHz, 30 min/25 kHz, control/45 kHz, 15 min/45 kHz, and 30 min/45 kHz (HIU time/frequency of HIU). The equipment was set in dynamic mode, which allowed the alternate “sweep” and “pulse” functions to work automatically to increase the ultrasound performance by 20% and ensure a uniform distribution of the acoustic fields. The initial sonication temperature was 4 °C (before HIU) and the final temperature after HIU treatment was 7.1 ± 0.2 and 7.9 ± 0.2 °C with treatment times of 15 and 30 min, respectively. Ultrasonicated milk and no ultrasonicated control milk were immediately used to make Oaxaca cheese.

### 2.2. Oaxaca Cheese Making Process

**Figure 1:**
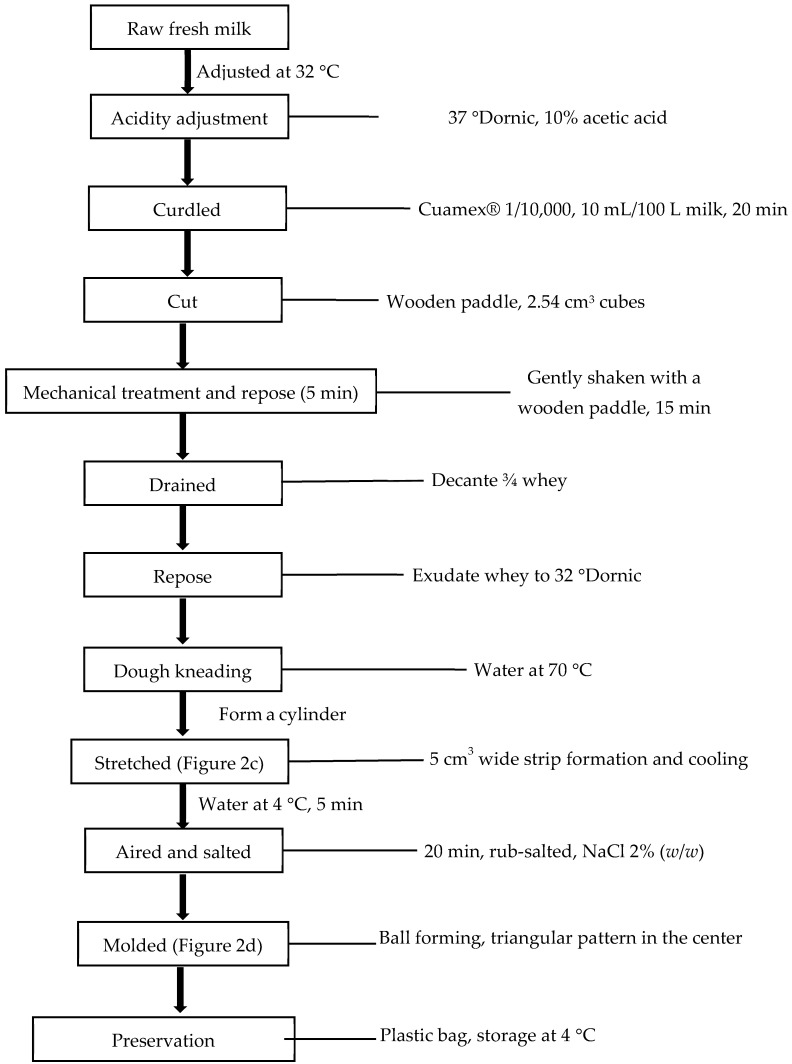
Flow chart for the production of Oaxaca cheese.

Oaxaca cheese was made in an artisanal way according to the methodology of Villegas [25]. The processing time elapsed since the milk reception until the cheese packaging was approximately 4 h. The procedure is described in Figure 1. For each treatment, 5 L of milk was used. Immediately after ultrasonication, the temperature of milk was increased by heating to 32–34 °C and the acidity was adjusted to 37 °D (pH 5.8–5.9) using glacial acetic acid diluted in water (10% *v*/*v*). Microbial rennet (Cuamix^®^, CHR Hansen A/S, Hørsholm, Denmark) previously diluted in water (10% *v*/*v*) was added and gently dispersed using a wooden paddle. After 20 min, the curd was coarsely cut with the wooden shovel to form cubes of 2.54 ± 0.6 cm^3^. The curd cubes were gently shaken for 15 min and allowed to stand for 5 min. Afterwards, 3/4 parts of the whey were removed by decanting and the curd was allowed to stand for approximately 30–45 min until the acidity of the exuded whey reached 32 °D. Then, the curd was cut with a wooden shovel into 2–3 cm^3^ cubes and the paste was hand-kneaded using hot water at 70 ± 2 °C, forming a cylinder that was later stretched to form an approximately 5 cm^3^ wide strip or thin ribbon, which was placed in cold water (4 °C) for 5 min. Finally, with the rubbed-salted ribbon (2% *w*/*w*), a traditional ball of Oaxaca cheese was formed following a triangular pattern in the center, which was placed in a plastic bag to be stored at 4 °C for 24 h. At this point, the cheese would have been ready to be consumed or marketed.

### 2.3. Physicochemical Evaluations and Real Cheese Yield

Moisture [26], protein [27], and fat [28] content in the cheese was determined 24 h after production. Color space was determined using the CIE (Commission Internationale de l’Éclairage) L*, a*, and b* parameters, where L* is lightness, a*(-) is greenness, and b*(+) is yellowness. Measurements were obtained with a colorimeter (Konica Minolta, CR 400, Tokyo, Japan) according to the CIE reference system. Three readings were taken for each sample in different areas of the same cheese and the averages were taken. Chroma (C*) and Hue angle (H*) were calculated by the expressions C* = √a*^2^ + b*^2^ and H* = tan^−1^ (b*/a*), respectively. The pH was measured with a pH meter (Sentron, Model 1001, Woonsocket, RI, USA). Measurements were taken by inserting the electrode directly into the cheese to a depth of 2 cm. Three readings were taken in three different areas of the sample and the average was obtained. The cheese yield was estimated after 24 h at 4 °C, considering the final weight of the cheese obtained in relation to the weight of the processed milk (volume × density). The yield was expressed in kg of cheese/100 L of milk.

### 2.4. Microbiological Analysis

Ten grams of each cheese was aseptically homogenized in 90 mL of phosphate buffer (APHA, pH 7.2, NutriSelect^®^Plus, Germany) and mixed with a Stomacher (Lab Blender, London, UK) at a maximum speed of 2 min. The homogenized sample was serially diluted (1:10) in a sterile phosphate buffer according to Mexican Official Standards [29]. Each dilution was plated onto specific media by triplicate. Psychrophiles and aerobic mesophilic microorganisms were plated onto plate count agar (PCA; Oxoid, Basingstoke, U.K.) and incubated at 5 °C ± 2 °C for 168 h and 35 ° C ± 2 °C for 48 ± 2 h, respectively [30,31]; lactic acid bacteria (LAB) were incubated anaerobically at 30 °C for 120 h (MRS stands for deMan, Rogosa and Sharpe, Oxoid, Basingstoke, U.K.); coliforms, *Escherichia coli* and *Staphylococcus aureus* counts were determined using Petrifilm according to the 3M™ instructions: Coliform/*E. coli* Count Plates, 3M™ Petrifilm™ Staph Express Count (STX) Plate and Disk, and incubated at 35 °C ± 1 °C for 48 h ± 2 h and 37 °C ± 1 °C for 24 h ± 2 h, respectively; potato dextrose agar (PDA; BD Bioxon Becton Dickinson and Company, Mex) acidified with 10% tartaric acid (Merck, Darmstadt, Germany) at 25 °C for molds-yeasts during 120 h. Culture and identification of *Salmonella* spp. was performed following the Mexican Official Standards [32]. Media were inoculated by spreading 100 µL of diluted samples onto the agar. Colony-forming units (CFU) were counted on the plates, with numbers ranging between 10 and 200 CFU. All determinations were conducted in triplicate. Numbers of bacteria were transformed from CFU mL^−1^ to log units (log_10_).

### 2.5. Empirical Stretchability Test

A ribbon was untangled from the ball of Oaxaca cheese and a rectangle of 5 cm × 1 cm (approximately 5 g) was cut out from it. The piece of cheese was subjected to a tension force by pulling with fingers in a direction parallel to the formation of the strands, in order to quantify the number of threads formed. The pulling force must be carefully controlled to avoid the breaking of the cheese strands. Subsequently, photographs were taken to register the formation of threads in the samples.

### 2.6. Statistical Analysis

Physicochemical and microbiological data were analyzed in a completely randomized block factorial design with the SAS 9.4 program (SAS Institute Inc., Cary, NC, USA), where the blocks were the milkings. Reported values are means ± standard deviation in which the differences between samples were determined using Tukey test (significance levels *p* < 0.05). The first factor was the ultrasound treatment time (5 and 10 min), and the second one was the ultrasound frequency (25 and 45 kHz), resulting in a complete factorial experiment with two controls, 0 min HIU/25 kHz and 0 min HIU/45 kHz.

## 3. Results and Discussion

### 3.1. Physicochemical Properties

**Figure 2:**
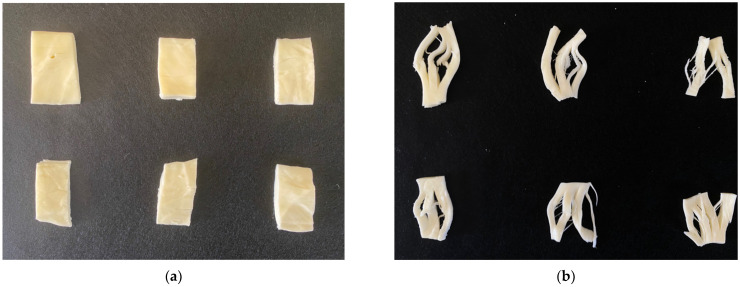

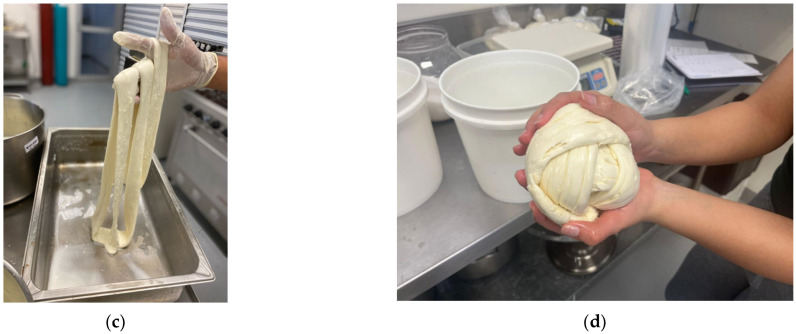
Images of Oaxaca cheese showing differences in appearance (**a**) and in the formation of strings or “threads” (**b**) due to the effect of ultrasound treatment (0, 15, and 30 min of HIU, 25 or 45 kHz, left to right and top to bottom); stretched (**c**) and ball forming or molded (**d**).

Ultrasound frequency had no effect on Oaxaca cheese color parameters (*p* > 0.05, Table 2, Figure 2a), but the ultrasonication time caused a significant increase in cheese lightness (*p* < 0.05, from 91.1 to 92.6 and 93.7 for 15 and 30 min of HIU, respectively). The greenness was higher in the ultrasonicated samples, and this parameter is relevant because the chroma is calculated from it. The main pigments in milk are: riboflavin, a green compound present in the aqueous phase; B-carotene, responsible for the yellow color, and; in a lesser extent, lutein [33]. However, no significant differences were found in the cheese tone or chroma (Figure 2a). Interestingly, significant differences were found between blocks (*p* < 0.05), meaning that not all blocks evaluated produced the same effect on the color of Oaxaca cheese. Cheeses from block one had greater luminosity, while cheeses from blocks two and three showed greater yellowness, brightness and were more colorful (greater chroma). Consequently, the hue value for the cheeses in block 1 was closer to the yellow color (90°). The fresh raw milk used in each repetition (block) came from different milkings, so it was expected to find significant difference between blocks. However, the ultrasound treatment seems to homogenize and reduce these color differences, since no significant differences were found in the cheese tone or chroma due to HIU frequency or time. Our results differ from previous results of lightness [18], in which yellow tones in Panela cheese made with ultrasonicated milk were not significantly different from those of the control (probe system, 400 W, 24 kHz, 10 min). The change in cheese color might be associated with the change in milk color due to HIU application, since the milk carotenoids are transferred to the cheese with minimal losses. Yellowness and lightness are the coordinates that are most frequently modified when using powers greater than 400 W and frequencies of 19–24 kHz [34]. Independently of the variations in the ultrasonication time or power, the size reduction of fat globules and a higher homogenization in liquid and semiliquid dairy are common effects of applying this range of ultrasonication frequencies. The change in color parameters of milk and milk-based products is apparently promoted by variations in ultrasonication power. The use of a power greater than 400 W seems to promote changes in chroma or a* coordinates. In this regard, Bermúdez-Aguirre and Barbosa-Cánovas [23] reported a significant higher lightness of fresh cheese made with thermosonicated milk (400 W, 24 kHz, 63 or 72 °C, 15 s to 30 min). In the present study, the ultrasound treatment is not enough to modify the fat globules. The changes in milk color are associated to the capacity of fat globules and casein micelles to disperse light. Therefore, the effects of ultrasound on the physical structure of milk (smaller size of fat globules and its release of triacylglycerides, cholesterol and phospholipids), cause a change of the light dispersion and milk color [35]. Moreover, the sonication temperature and the ultrasound system significantly influence the size reduction of the fat globule. Sonication powers higher than 30 W and temperatures higher than 50 °C, reduced fat globule size from 3.39–3.89 μm to 0.37–1.9 μm [36]. According to Nozière et al. [33], milk carotenoids are transferred to milk with minimal loss and thus contribute to the yellow cheese coloring.

Regarding the quantitative analysis of the constituents, the ultrasound frequency had no effect on moisture content (*p* = 0.8777), protein (*p* = 0.135), fat (*p* = 0.8741) or pH (*p* = 0.4576) of Oaxaca cheese (Table 3). However, an increase in frequency (from 25 to 45 kHz) produced a significant lower ash content (from 2.08 to 1.84%, *p* = 0.0043) and higher yield (from 12 to 13 kg/100 L) of milk, (*p* = 0.001). Regarding the treatment time, the results showed that the yield and protein contents are significantly higher in samples treated with HIU (*p* < 0.0001 and *p* = 0.0114, respectively), regardless of treatment time (15 or 30 min).

**Figure 3:**
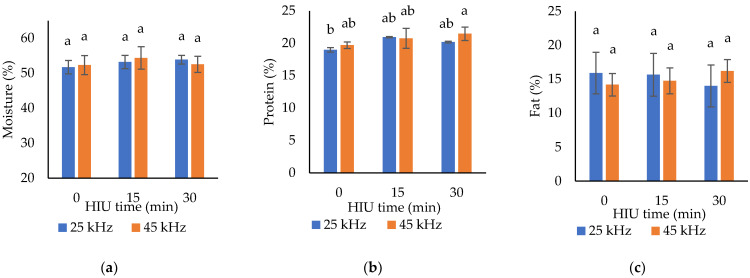

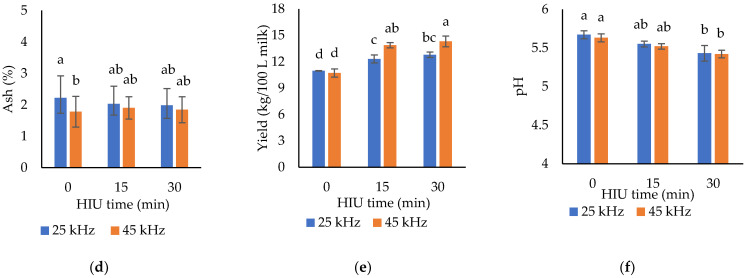
Effects of HIU frequency × HIU applied time to fresh raw milk on the pH (**a**), yield (**b**), and moisture (**c**), protein (**d**), fat (**e**) and ash (**f**) content of Oaxaca cheese. Data are expressed as mean ± standard deviation (*n* = 3). ^a,b,c,d^ Different letters in the columns within the same graph indicate significant differences between treatments (Tukey´s multiple range tests assuming a significant difference at *p* < 0.05).

The highest yield was obtained when long ultrasonication times (30 min) were used on the milk. Compared to the control (fresh untreated raw milk), 25.93% more Oaxaca cheese was produced when milk was HIU-treated (30 min HIU). As shown in Figure 3e, the combination of factors was significant (*p* < 0.0001), and the highest yield was obtained with the 45 kHz and 25 kHz combinations with 30 min of HIU. According to Table 3, the higher yielding in cheeses made with ultrasonicated milk is due to the greater protein content, which was 1.4 and 1.5% higher after HIU treatment for 15 and 30 min, respectively, compared to the control. The highest protein content in Oaxaca cheese was obtained with the milk treated with combined 45 kHz and 30 min of HIU (Figure 3b). Although, the improvement of cheese yield obtained with 25 kHz and 30 min of HIU, seems to be associated with the increment in moisture content when using this combination of factors (Figure 3a).

The cheeses obtained with ultrasonicated milk tend to have a greater moisture content, as the resulting gels might have a higher water retention capacity due to a more hydrophilic surface. Whey proteins are incorporated into the surface of casein micelles when cheese is made with heat-treated milk [37], so the cavitation effect produced by ultrasound could have favored the greater protein content in cheeses made with sonicated milk. Low sonication temperatures (20 kHz, 450 W, probe system, amplitude 50%, 1–60 min, 6 ± 4 °C), produce minor changes in whey protein that should not be disregarded. Prolonged sonication augments the denaturing enthalpy due to protein aggregation and leads to slight changes in the secondary structure and hydrophobicity of proteins [38]. According to Gregersen et al. [36], the ultrasound treatment (20 kHz, 27–70 °C, 10–50 W) in whole milk homogenizes fat globules and causes acid-induced changes in gelification. Although the high temperature on its own was not sufficient to induce significant denaturing of whey proteins, a greater association of caseins to fat globules was observed, possibly determined by the acidity changes. Although the combination of factors was not significant for fat content (Figure 3c), this component might have contributed to the high cheese yield obtained with 45 kHz and 30 min of HIU, which had the highest fat content.

Carrillo-Lopez et al. [18] reported no changes in the fat content of Panela cheese made with ultrasonicated milk. Jalilzadeh et al. [22] also found no differences in the fat content of Iranian feta cheese matured for 60 d and made with ultrasound (20–60 kHz, 20 min) treated milk. The higher yield in fresh cheese has been documented by Bermúdez-Aguirre and Barbosa-Cánovas [23], who observed a gain of up to 100% in yield when milk at 63 °C was treated with ultrasound for 30 min. These researchers attributed this yield to the higher moisture content as the ultrasound treatment time increased, so that the increase in moisture content was possibly due to the reorganization of the molecules in the cheese to favor water retention.

Lower temperatures (42 °C) in ultrasound processing of raw milk produce quick gelification times and firmer curds, compared to ultrasound treatments at 54 °C. The greater strength of the enzymatic curd was attributed to protein–protein and protein–lipid interactions as an effect of the ultrasound [39]. The curd matrix was denser, with more irregular clustered strands and submicron lipid droplets embedded within the protein strands. Ultrasound treatment accelerates the firmness of the curd so that a harder curd is obtained when proteolytic enzymes, including chymosin and pepsin, are incorporated [40]. Cheese yield is associated with coagulation firmness, so a firm curd increases yield, improves texture, and reduces protein loss in cheese [18]. These authors showed significant differences of up to 3.79% more protein content in cheeses made with ultrasonicated milk compared to those made with fresh raw milk. Ultrasound is known to induce structural changes in milk proteins and modify the surface hydrophobicity of proteins, leading to the formation of firm micellar casein gels with low syneresis [10,11]. The effect of sonication depends on the presence and state of the fats (entry milk temperatures and treatment temperatures); therefore, the fact that Liu et al. [11] used skimmed milk must be considered, whereas Chandrapala et al. [10] used pasteurized milk and gel formation was not evaluated. The structural changes produced by ultrasound favored the denaturation of the caseins, which were aligned and oriented in fibers due to the effect of mechanical work (kneading). Cheeses with higher protein content (HIU 15 min, 25 or 45 kHz, and HIU 30 min/25 kHz) formed a greater number of “strings” during the empirical thread formation test (Figure 2b).

The higher water-retention capacity and moist content in cheese as a result of ultrasound treatment has been reported by Jalilzadeh et al. [22]. Protein content results vary along the sonication conditions. For instance, Jalilzadeh et al. [22] and Hayaloglu et al. [13] observed lower protein content during storage, whereas in previous studies we have found higher protein content when using HIU [18]. This effect could be explained by the sonication temperature, which affects the characteristics of the curd and the interaction of its components [39]. This explains the higher protein and moisture content observed in cheeses made with ultrasonicated milk. Although significant differences were found between blocks for humidity, fat, and ashes, the ultrasound treatment seems to reduce these differences, since no significant differences were found in these response variables due to the effect of the frequency or time of HIU, except in the ash content (Figure 3d), which seems to be related to the demineralization of the casein micelle by acidification.

Acidification constitutes a process of protein denaturation that causes the demineralization of the micelles (the caseins unfold and begin to precipitate) [25]. The pH value decreased significantly (*p* = 0.0005) in cheeses made with ultrasonicated milk (from 5.65 in controls to 5.42–5.54 in cheeses made with HIU-treated milk). This can be seen in more detail in the combination of factors shown in Figure 3f. The pH was significantly reduced as the ultrasound treatment time increased (*p* = 0.0037). *Pasta filata* cheeses, including Oaxaca cheese, have the particularity that the acidified curd is kneaded with hot water to plasticize and stretch it, forming aligned band structures that separate like “threads” [2].

Acidification (up to pH 5.5–5.6) is the most important critical point during manufacturing [41]. According to Table 3, all the pH values are within the normal pH range characteristic for this type of cheese. The subsequent acidification (24 h of storage at 4 °C) as the ultrasound treatment time increased seems to be associated with the promotion of microbial activity, particularly, that of lactic acid bacteria, considering the fresh raw milk as starting material.

Carrillo-Lopez et al. [18] also observed a significant reduction in the pH of cheese made with fresh raw milk as the HIU treatment time increased. Jalilzadeh et al. [22] also observed the same trend in Iranian feta cheese after 60 d of storage. In their study, the activity of lactic acid bacteria reduced the pH value at the end of the storage period [13]. In addition, Villamiel et al. [40] observed a decrease in pH when fresh milk was treated with ultrasound (24 kHz, 400 W). The decrease in the pH of milk seems to be associated with the increase in the enzymatic activity of proteases and other native milk enzymes on triacylglycerides due to the effect of cavitation, producing the hydrolysis of phosphoric esters and the release of fatty acids into the milk in addition to the formation of some compounds such as nitrites and nitrates as well as hydrogen peroxide and other free radicals [13,14,15,16].

### 3.2. Microbiological Evaluations

**Figure 4:**
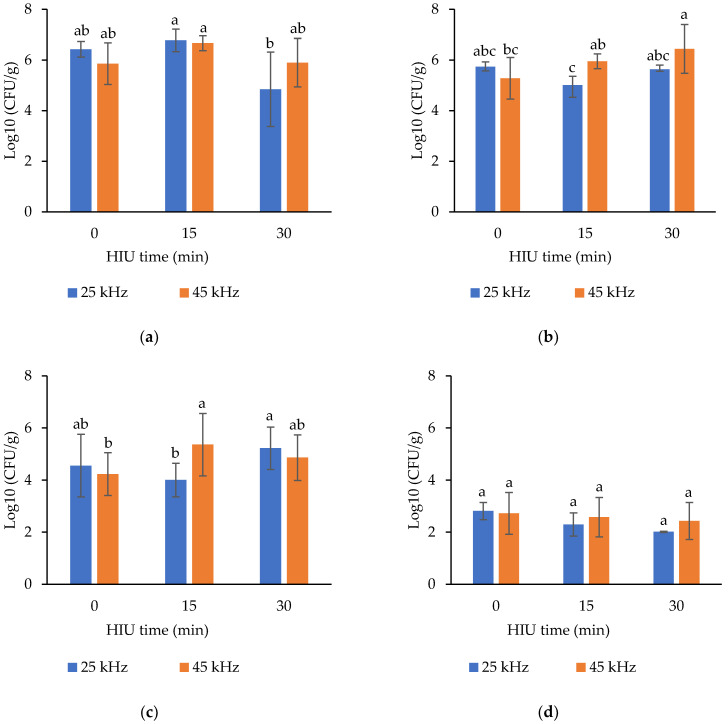
Effects of HIU frequency × HIU applied time to fresh raw milk on the mesophilic bacteria (**a**), psychrophilic bacteria (**b**), lactic acid bacteria (**c**), and mold and yeasts (**d**) counts of Oaxaca cheese. Data are expressed as mean ± standard deviation (*n* = 3). ^a,b,c^ Different letters in the columns within the same graph indicate significant differences between treatments (Tukey´s multiple range tests assuming a significant difference at *p* < 0.05).

As shown in Table 4, ultrasound application results in significantly higher counts of mesophilic bacteria (*p* = 0.013) when short treatment times (15 min) were used, regardless of the frequency used (Figure 4a). The lowest counts were obtained when long treatment times (30 min) at frequencies of 25 kHz were used (Figure 4a). Significant differences were also observed between blocks (*p* = 0.0252), with a lower total aerobic load count in the cheeses from block 1. This could suggest that there were differences in the hygienic quality of the fresh raw milk used in each block (batch).

According to Mexican regulations [18], the mesophilic bacteria count allowed in cheese is ≤5 log_10_ CFU/g, so cheese made with ultrasonicated milk at 25 kHz for 30 min complies with current regulations by having counts of 4.85 log_10_ CFU/g (Figure 4a). Nevertheless, these results must be considered cautiously, since the standard deviation value large and the individual values could be higher than 5 log_10_ CFU/g. The significantly higher mesophilic bacteria count in cheeses made with milk treated for 15 min seems to be associated with the stimulation of the growth of this group of bacteria by the effect of ultrasound treatment, which increases the speed of oxygen and nutrient transport to bacterial cells. In this case, the ultrasound used was in the threshold between low and high intensity (70 kHz, 2–4 W/cm^2^, 30 min) and was employed to eliminate bacteria adhered to high density polyethylene surfaces [42]. As the ultrasound treatment times and intensities in hoax’s milk extend (from 5 to 15 min, 20 °C, 430 and 338 W), the aerobic bacterial counts are significant [20]. Short ultrasonication times (200 W, 24 kHz, 15–25 °C, 2, 4, 8 and 16 min) result in higher viable counts in milk during storage [21].

Furthermore, the increase in mesophilic flora during coagulation and whey drainage could be due to multiplication of bacteria such as lactic acid bacteria. Thermosonication has been reported to have a positive effect on the reduction of microorganisms in fresh raw milk. In this regard, Herceg et al. [43] reported significant inactivation of mesophilic bacteria in fresh raw milk only when using ultrasound treatment (20 kHz, 6, 9, and 12 min) duration of 12 min combined with high temperature (60 °C) using a probe system (60, 90 and 120 μm amplitude). With ultrasonicated fresh raw milk at 20 °C, these researchers reported up to 1.52 log_10_ more CFU/mL, compared to controls without sonication, which presented counts of 6.34 log_10_ CFU/mL, values close to those obtained in the present investigation in untreated cheese.

Similar results were obtained by Ganesan et al. [44], who showed lower counts of this group of bacteria by up to 5 log_10_ CFU/mL when using 72 °C during the ultrasonication (probe system, 216 μm) of fresh raw milk and reducing the treatment time from 15 s (flash pasteurization) to 10 s (with thermosonication). According to Balthazar et al. [19], ultrasound treatment significantly decreases mesophilic bacteria counts in stored semi-skimmed sheep’s milk (1–6 months), provided that two sonication cycles are used and that the temperature range reached during the process is between 40 and 69 °C using a probe system (78 or 104 W, 4–8 min). In addition to temperature, other factors can influence the reduction of the microbial load in milk. For example, an increase in power and exposure time to ultrasound significantly decreases the counts of mesophilic bacteria in buffalo milk because cavitation can damage the cell wall of microorganisms when the bubbles implode, generating high rates of micro shear [20]. Similar results were obtained in the present study where an increase in sonication time of the fresh raw milk (from 15 to 30 min) produced significant lower counts of mesophilic bacteria in cheese.

Psychrophilic bacteria counts (Table 4) decreased significantly at low frequencies (25 kHz, *p* = 0.0067). Long times of ultrasound treatment produces higher counts of this group of bacteria (*p* = 0.013). The best combination for the control of this group of bacteria was that of 25 kHz and 15 min of HIU (Figure 4b). According to Chouliara et al. [21], ultrasound treatment is beneficial when treatment times of 16 min are used in milk. These researchers found that psychrotrophic bacterial counts (from 5.68 log_10_ CFU/mL in non-HIU controls to 3.51 log_10_ CFU/mL with 16 min of HIU) are reduced immediately after HIU treatment. However, a more effective reduction can be achieved when ultrasound is combined with thermization (55 °C, 15 s) or pasteurization (75 °C, 15 s). Similar results were obtained in the present study, since the counts of psychrophilic bacteria decreased significantly when 15 min of treatment with HIU and 25 kHz was used. The higher counts in this bacterial group resulting from longer treatment times (30 min) could be associated with the release of nutrients and growth stimulation [18]. Van Hekken et al. [39] also observed lower mesophilic and psychrophilic bacteria counts in ultrasonicated raw milk and stored for 48 h. According to these authors, temperatures of 54 °C during sonication significantly reduced the number of psychrophilic bacteria in raw milk, making HIU an effective technology to reduce spoilage microorganisms in refrigerated dairy products.

In the present study, high frequencies (45 kHz) resulted in significantly higher counts of psychrophiles, with no effects upon molds and yeasts counts, opposed to Jalilzadeh et al. [22], who did not observe significant differences in *Penicillium chrysogenum* mold counts (strain with psychrotrophic characteristics) in Iranian feta-type cheese when different ultrasound frequencies were used (20, 40, and 60 kHz, 20 min). Although in their study, the sonication was not carried out on raw milk but on ultrafiltered pasteurized milk which was then inoculated with *P. chrysogenum*.

The results in Table 4 indicate that the 25 kHz frequency was highly efficient in reducing the counts of coliforms (*p* = 0.0239). The treatment times had no effect on the observed counts (*p* = 0.7074) and the combination of factors was not significant (*p* = 0.7074). The best combinations were those of 25 kHz with 0, 15, and 30 min of HIU, with counts of 0 log_10_ CFU/mL, so we cannot attribute the low microbial load to HIU per se. The blocks had no significant effect on the coliform bacteria counts in Oaxaca cheese (*p* = 0.0845, Table 4), but provide an idea of the difference in the hygienic quality of the milk used in each of the batches (blocks).

Similar to mesophilic bacteria, cheeses from block 1 had the lowest coliform counts (0 log_10_ CFU/mL) and block 3 showed the highest counts. According to the current Mexican regulations [18], the highest permissible count is ≤1 log_10_ CFU/g for coliform bacteria. According to this, all the cheeses produced comply with the standard, even the controls without HIU application. The kneading of the paste with hot water (75 °C) as part of the cheese making process could have had a significant influence on the hygienic quality assurance of the evaluated cheeses. In artisan cheeses made with raw milk, coliform bacteria counts above the norm have been reported. For instance, Silva-Paz et al. [45] observed coliform counts above 2.1 log_10_ CFU/g in 95% of the cheeses from the Ojos Negros region in Mexico.

It is important to consider that high microbial loads (mesophilic and coliform bacteria) do not necessarily indicate contamination by fecal matter, but low hygienic quality due to poor handling practices during milking and milk storage. Several authors have found relevant effects of ultrasound in the decontamination of milk and dairy products. In previous studies, we have reported significant reductions in total coliform counts in Panela cheese made with fresh raw milk that was HIU-treated using a probe system and amplitudes of 50%, with no effect when using 100% amplitude [18]. In buffalo milk, Al-Hilphy et al. [20] also reported significant decreases in coliform bacteria counts as the exposure time and ultrasound intensity increased (5–15 min, 338 and 430 W, 20 °C). Balthazar et al. [19] also reported significant decreases in coliform counts in skim milk, but under thermosonication cycles with temperatures between 40 and 69 °C (78 or 104 W, 4–8 min). Results similar to ours were obtained by Engin and Yuceer [46], who reported the inefficiency of the HIU for the microbial control of coliforms in raw milk (20 kHz, 5 °C, 15 min).

The ultrasound frequency had no significant effect on the counts of lactic acid bacteria (Table 4) present in Oaxaca cheese (*p* = 0.1909). However, the counts of this group of bacteria were significantly higher when using long treatment times (30 min, *p* = 0.0236), regardless of the frequency (25 or 45 kHz, Figure 4c). Our results are similar to those obtained by Balthazar et al. [19], who reported that the counts of some genera of lactic acid bacteria such as lactobacilli and streptococci are dependent on the sonication temperature. At low temperatures during the two sonication cycles (40 and 52 °C), the counts of lactobacilli and streptococci are preserved and even increase after a storage period of 7 d. Ultrasound treatment of Ewe’s milk (78 and 104 W, 8 and 4 min) could be advantageous, since it preserves LAB and inactivates contaminant bacteria in the frozen milk [19], whereas pasteurization completely eliminates Lactobacilli and lactic Streptococci.

While the frequency had no effect on the counts of molds and yeasts (Table 4), ultrasound times of 15 and 30 min (*p* = 0.0355) produced significantly lower counts of this group of microorganisms. The combination of factors was not significant (*p* = 0.083, Figure 4d), however the lowest counts were obtained with 25 kHz and 30 min of HIU. Block 2 had the highest counts of molds and yeasts (*p* = 0.0007). The inactivation of molds and yeasts has only been reported when ultrasound is used in combination with high temperatures (thermosonication). For example, Guimarães et al. [47] reported the inactivation of these microorganisms in a prebiotic whey drink treated with ultrasound (200–600 W, 19 kHz, 53 °C, 3 min). Jelicic et al. [24] also reported the inactivation of molds and yeasts in whey of rennet cheese treated with thermosonication (240–400 W, 35–45 °C).

Consistent with our results, Marchesini et al. [48] observed that short ultrasound times (50, 100, 200, and 300 s, amplitude 70 and 100%) preserve the structural and functional integrity of the yeast *Debaryomyces hansenii*, which is naturally found in milk and promotes milk gelation.

Only block 3 had a significantly higher count (*p* < 0.0001) of *S. aureus*; however, non-significant differences were found between frequencies or between times of HIU. This suggests poor hygienic quality of the milk in this block, or recontamination after dough kneading, as in the case of mesophile bacteria (Table 4). The combination of factors was not significant (*p* = 0.4651).

For *Salmonella* spp. and *E. coli*, no positive samples were found in the evaluated cheeses. According to the Mexican standard NOM-243-SSA-2010 [29], the maximum limit allowed for *S. aureus* in fresh cheeses is 3 log_10_ CFU/g, so all the cheeses evaluated comply with the regulations. The same standard establishes maximum limits for *E. coli* of 2 log_10_ CFU/g and the absence of *Salmonella* spp., and our results indicated the absence of such pathogenic bacteria in Oaxaca cheese, even in the control. Thus, it is inferred that mixing the dough with hot water at 75 °C guarantees the inactivation (assuming that they were present in the milk) of *S. aureus, E. coli* and *Salmonella* spp., ensuring the safety of Oaxaca cheese made with fresh raw milk without constituting any risk for consumers.

Silva-Paz et al. [45] also reported absence of *Salmonella* spp. in artisanal cheese made with raw milk. However, the inactivation of pathogenic bacteria in milk and its products generally involves the combination of ultrasound with other accompanying technology. Shamila-Syuhada et al. [49] reported that the inactivation of *Salmonella enterica* ssp., *Salmonella tiphymurium*, *S. aureus* and *E. coli* in milk is possible in the presence of H_2_O_2_ or the active lactoperoxidase system, since HIU alone does not ensure microbial quality and milk safety. Engin and Yuceer [46] achieved reductions as low as 1.5 and 0.77 log_10_ CFU/mL in cow’s milk treated with ultrasound for 15 min at 5 °C. Moreover, the combined application of heat with ultrasound has generated promising results. The inactivation of pathogenic bacteria is achieved when ultrasound times of 12 min, high temperatures (60 °C) and amplitudes (120 μm) are combined; however, it has been observed that Gram-negative bacteria such as *E. coli* are more susceptible to ultrasound treatment than Gram-positive (*S. aureus*) [43].

## 4. Conclusions

According to the physicochemical analysis, cheeses made with ultrasonicated milk have a better appearance, have high yields, and a greater protein content than cheeses conventionally manufactured. Thus, they could be highly competitive in the cheese industry. Although ultrasonication at low temperatures is effective, additional studies simultaneously using ultrasound combined with other technologies are needed to efficiently reduce bacterial groups (mesophiles and psychrophiles) that cause the detriment of the product. The hygienic quality of the raw milk prior to the ultrasound treatment and microbial quality surveillance along the product’s shelf-life should also be considered in future experiments, in order to assure the scalability of the process to the industrial level.

This study proposes an alternative for the preservation of genuine Oaxaca cheese, with the specific features conveyed by using raw milk, thus, avoiding the loss of historical roots of the Mexican culture.

## Figures and Tables

**Table 1:** Physicochemical parameters of fresh raw milk prior to ultrasound treatment.

Block	Titratable Acidity (°Dornic)/pH	Fat (%)	Protein (%)	Lactose (%)	Non-Fat Solids (%)
1 2 3	17/6.56 17/6.57 17/6.57	3.64 3.57 3.59	3.19 3.22 3.18	4.91 4.95 4.89	8.81 8.88 8.78

**Table 2:** Effects of blocks (BLO), HIU (high-intensity ultrasound) frequency, HIU applied time and HIU frequency × HIU applied time on fresh raw milk on the color space CIE L* (lightness) a* (greenness) b* (yellowness) of Oaxaca cheese.

Treatment	CIE L*a*b*				
BLO	L*	a*	b*	Hue	Chroma
1 2 3	91.8 ± 1.6 ^b^	−3.09 ± 0.1 ^a^	15.5 ± 1.0 ^a^	101.3 ± 0.4 ^b^	15.8 ± 0.9 ^a^
	94.6 ± 1.1 ^a^	−3.18 ± 0.3 ^a^	15.3 ± 0.5 ^a^	101.2 ± 0.3 ^b^	15.6 ± 0.5 ^a^
	91.0 ± 2.2 ^b^	−3.11 ± 0.2 ^a^	14.2 ± 0.7 ^b^	102.4 ± 0.8 ^a^	14.5 ± 0.7 ^b^
HIU frequency (kHz)	L*	a*	b*	Hue	Chroma
25 45	92.6 ± 1.7 ^a^	−3.11 ± 0.2 ^a^	14.9 ± 0.8 ^a^	101.4 ± 0.5 ^a^	15.2 ± 0.9 ^a^
	92.3 ± 2.8 ^a^	−3.14 ± 0.3 ^a^	15.1 ± 1.0 ^a^	101.8 ± 0.9 ^a^	15.4 ± 0.9 ^a^
HIU time (min)	L*	a*	b*	Hue	Chroma
0 15 30	91.1 ± 2.5 ^b^ 92.6 ± 1.6 ^a,b^ 93.7 ± 2.0 ^a^	−3.39 ± 0.2 ^b^ −2.98 ± 0.0 ^a^ −3.0 ± 0.1 ^a^	14.8 ± 0.8 ^a^ 15.1 ± 1.1 ^a^ 15.1 ± 0.9 ^a^	101.7 ± 0.6 ^a^ 101.5 ± 0.6 ^a^ 101.6 ± 1.1 ^a^	15.1 ± 0.7 ^a^ 15.4 ± 1.1 ^a^ 15.4 ± 0.9 ^a^
HIU frequency × HIU time					
25 kHz					
0 min	91.9 ± 1.9 ^a^	−3.33 ± 0.0 ^ab^	15.3 ± 0.7 ^a^	101.9 ± 0.6 ^a^	15.6 ± 0.7 ^a^
15 min	92.5 ± 1.5 ^a^	−2.97 ± 0.1 ^a^	14.7 ± 0.9 ^a^	101.3 ± 0.6 ^a^	15.0 ± 0.9 ^a^
30 min	93.4 ± 2.0 ^a^	−3.02 ± 0.1 ^ab^	14.6 ± 1.1 ^a^	101.1 ± 0.6 ^a^	14.9 ± 1.1 ^a^
45 kHz					
0 min	90.3 ± 3.3 ^a^	−3.45 ± 0.3 ^b^	14.2 ± 0.5 ^a^	101.5 ± 0.6 ^a^	14.6 ± 0.5 ^a^
15 min	92.6 ± 2.0 ^a^	−2.99 ± 0.0 ^a,b^	15.6 ± 1.2 ^a^	101.8 ± 0.6 ^a^	15.9 ± 1.2 ^a^
30 min	94.0 ± 2.5 ^a^	−2.98 ± 0.2 ^a,b^	15.5 ± 0.6 ^a^	102.2 ± 0.6 ^a^	15.8 ± 0.6 ^a^

^a,b^ Different letters within the same column indicate significant differences between treatments (Tukey’s multiple range tests assuming a significant difference at *p* < 0.05). Data are expressed as mean ± standard deviation (*n* = 3).

**Table 3:** Effects of blocks (BLO), HIU (high-intensity ultrasound) frequency, and HIU applied time to fresh raw milk on the moisture, protein, fat and ash content, actual yield (kg cheese/100 L milk), and pH of Oaxaca cheese.

Treatment	Component Analysis, Actual Yield and pH					
BLO	Moisture (%)	Protein (%)	Fat (%)	Ash (%)	Yield	pH
1 2 3	51.6 ± 1.5 ^b^	20.7 ± 1.5 ^a^	16.8 ± 1.0 ^a^	2.26 ± 0.3 ^a^	12.4 ± 1.7 ^a^	5.53 ± 0.1 ^a^
	54.7 ± 1.9 ^a^	20.3 ± 0.6 ^a^	13.1 ± 0.5 ^b^	1.38 ± 0.1 ^b^	12.5 ± 1.6 ^a^	5.54 ± 0.2 ^a^
	52.7 ± 1.9 ^ab^	20.0 ± 0.9 ^a^	15.6 ± 0.7 ^ab^	2.24 ± 0.2 ^a^	12.6 ± 1.2 ^a^	5.54 ± 0.1 ^a^
HIU frequency (kHz)	Moisture (%)	Protein (%)	Fat (%)	Ash (%)	Yield	pH
25 45	52.9 ± 1.8 ^a^	20.1 ± 0.9 ^a^	15.2 ± 2.8 ^a^	2.08 ± 0.5 ^a^	12.0 ± 0.9 ^b^	5.55 ± 0.1 ^a^
	53.1 ± 2.6 ^a^	20.7 ± 1.2 ^a^	15.1 ± 1.8 ^a^	1.84 ± 0.4 ^b^	13.0 ± 1.8 ^a^	5.52 ± 0.1 ^a^
HIU time (min)	Moisture (%)	Protein (%)	Fat (%)	Ash (%)	Yield	pH
0 15 30	52.0 ± 2.5 ^a^ 53.8 ± 1.6 ^a^ 53.2 ± 2.0 ^a^	19.4 ± 0.6 ^b^ 20.9 ± 1.0 ^a^ 20.8 ± 1.0 ^a^	15.1 ± 2.4 ^a^ 15.2 ± 2.4 ^a^ 15.1 ± 2.5 ^a^	2.0 ± 0.6 ^a^ 1.97 ± 0.4 ^a^ 1.91 ± 0.4 ^a^	10.8 ± 0.3 ^b^ 13.1 ± 0.9 ^a^ 13.6 ± 0.9 ^a^	5.65 ± 0.1 ^a^ 5.54 ± 0.0 ^b^ 5.42 ± 0.1 ^c^

^a,b,c^ Different letters within the same column indicate significant differences between treatments (Tukey´s multiple range tests assuming a significant difference at *p* < 0.05). Data are expressed as mean ± standard deviation (*n* = 3).

**Table 4:** Effects of blocks (BLO), HIU frequency and HIU applied time on the mesophiles, psychrophiles, coliform, lactic acid bacteria and molds and yeasts counts of Oaxaca cheese.

Treatment	Microorganism Groups				
BLO	Mesophiles (log_10_ CFU/g)	Psychrophiles (log_10_ CFU/g)	Coliforms (log_10_ CFU/g)	Lactic acid bacteria (log_10_ CFU/g)	Molds and yeasts (log_10_ CFU/g)
1 2 3	5.44 ± 1.4 ^b^	5.80 ± 0.7 ^a^	Not detected^a^	4.99 ± 0.8 ^a^	2.13 ± 0.2 ^b^
	6.16 ± 0.4 ^a,b^	5.55 ± 0.4 ^a^	0.33 ± 0.8 ^a^	5.42 ± 0.6 ^a^	3.06 ± 0.6 ^a^
	6.64 ± 0.5 ^a^	5.68 ± 0.5 ^a^	1.05 ± 1.2 ^a^	3.70 ± 0.5 ^b^	2.24 ± 0.3 ^b^
HIU frequency (kHz)	Mesophiles (log_10_ CFU/g)	Psychrophiles (log_10_ CFU/g)	Coliforms (log_10_ CFU/g)	Lactic acid bacteria (log_10_ CFU/g)	Molds and yeasts (log_10_ CFU/g)
25 45	6.0 ± 1.2 ^a^	5.46 ± 0.4 ^b^	Not detected ^b^	4.59 ± 1.0 ^a^	2.37 ± 0.4 ^a^
	6.1 ± 0.8 ^a^	5.89 ± 0.6 ^a^	0.92 ± 1.1 ^a^	4.82 ± 1.0 ^a^	2.58 ± 0.7 ^a^
HIU time (min)	Mesophiles (log_10_ CFU/g)	Psychrophiles (log_10_ CFU/g)	Coliforms (log_10_ CFU/g)	Lactic acid bacteria (log_10_ CFU/g)	Molds and yeasts (log_10_ CFU/g)
0 15 30	6.14 ± 0.6 ^a,b^ 6.72 ± 0.3 ^a^ 5.37 ± 1.3 ^b^	5.51 ± 0.3 ^b^ 5.48 ± 0.6 ^b^ 6.04 ± 0.5 ^a^	0.33 ± 0.8 ^a^ 0.38 ± 0.9 ^a^ 0.67 ± 1.0 ^a^	4.39 ± 0.9 ^b^ 4.68 ± 1.1 ^a,b^ 5.04 ± 0.8 ^a^	2.77 ± 0.6 ^a^ 2.44 ± 0.6 ^a,b^ 2.22 ± 0.5 ^b^

^a,b^ Different letters within the same column indicate significant differences between treatments (Tukey´s multiple range tests assuming a significant difference at *p* < 0.05). Data are expressed as mean ± standard deviation (*n* = 3).

## Data Availability

Data is contained within the article.
